# New record of *Anopheles superpictus* (Diptera: Culicidae) in central Italy (Tuscany): resting adults and evidence of natural breeding sites

**DOI:** 10.1186/s13071-025-06960-3

**Published:** 2025-08-02

**Authors:** Ilaria Bernardini, Francesco Severini, Michela Menegon, Claudia Mangiapelo, Riccardo Bianchi, Gioia Bongiorno, Marco Di Luca, Daniela Boccolini

**Affiliations:** Dipartimento Malattie Infettive, Reparto Malattie Trasmesse da Vettori, Istituto Superiore di Sanità, Viale Regina Elena, 299, 00161 Rome, Italy

**Keywords:** Malaria vector, Residual anophelism, Malaria surveillance

## Abstract

**Background:**

*Anopheles superpictus* (subgenus *Cellia*) plays an efficient role in malaria transmission in countries of the Mediterranean basin, Eastern Europe, and the Caucasus region, where it has been involved in the transmission of both *Plasmodium falciparum* and *P. vivax*. In Italy, this species was historically considered a secondary malaria vector, primarily recorded in the South including Sicily, along small rivers. It was less frequently observed in central Italy, only rarely reported in the North. Between 2022 and 2024, as part of routine investigations on residual anophelism, *An. superpictus* specimens were collected for the first time in the southern Maremma Plain (Magliano in Toscana, Grosseto, Tuscany).

**Methods:**

Adult mosquitoes were collected in animal shelters and tool premises of two farms using traps and manual aspiration methods. Larval sampling was performed by exploring potential breeding sites along the Albegna riverbed. Anophelinae adults and larvae were morphologically identified at the species level, and subsamples were molecularly confirmed by sequencing the cytochrome c oxidase subunit 1 gene and the ribosomal DNA internal transcribed spacer 2 region.

**Results:**

A total of 1,106 adult mosquitoes were collected from July to October 2022. *Anopheles superpictus* was the most abundant species recorded (*n* = 512; 46%), peaking in August and found in sympatry with *An. labranchiae*, the main historical malaria vector in Maremma Plain. *Anopheles superpictus* larvae were found in seven of the twelve investigated sites, with 50 specimens collected (mean density: 0.1–0.2 larvae per dip). Snapshot surveys in September 2023 and 2024 confirmed the stable occurrence of the species in the area.

**Conclusions:**

*Anopheles superpictus* was consistently recorded in an area where it had not been previously documented. These findings provide new insights into the current presence and distribution of the species in Italy and at the European level, notably including the first recent identification of natural breeding sites. Furthermore, the results highlight the importance of residual anophelism surveys in malaria surveillance, supporting the development of targeted control strategies and preparedness plans for potential malaria reintroduction in at-risk regions.

**Graphical Abstract:**

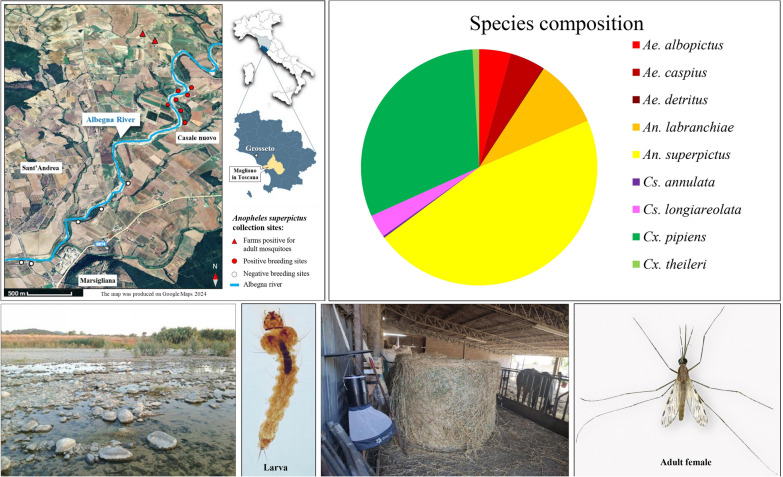

## Background

Malaria is a disease that still poses globally important challenges. In endemic countries, it continues to represent one of the more threatening public health concerns [1]. In non-endemic settings, it is the most frequently imported parasitic disease, and the persistence of putative vectors [2] underlines the need to implement and strengthen surveillance systems, particularly across European countries bordering the Mediterranean basin [3]. In Italy, *Plasmodium falciparum* transmission was virtually interrupted by 1948, just one year after the launch of the National Eradication Campaign. Nonetheless, sporadic *P. vivax* cases continued to be reported until 1962, and the country was officially declared malaria free in 1970 [4]. Former malaria vectors, such as *Anopheles labranchiae* Falleroni, 1926, *An. sacharovi* Favr, 1903, both members of the *An. maculipennis* complex (*Anopheles*) Meigen 1818, and *An. superpictus* (*Cellia*) Grassi 1899 are still present in scattered foci across the country. *Anopheles labranchiae* has been recorded along central-southern coastal areas, including Sicily and Sardinia [5–7]. *Anopheles sacharovi* historically restricted to parts of the Adriatic and Tyrrhenian coastlines and Sardinia, was presumed extinct since the late 1960s but was recently rediscovered in the Apulia region (Lecce province), where it had never been recorded during the malaria-endemic period [8]. The third taxon, i.e. *An. superpictus*, was historically considered a secondary malaria vector in Italy [9–11]. However, across its broad Palearctic distribution range, this species has instead played an efficient role as a malaria vector [12–14]. In the southeastern Mediterranean basin, including North Africa and West Asia, as well as in Eastern Europe and the Caucasian area of the Western Palearctic Region [15–17], it has been involved in the transmission of both agents of human malaria, *P. falciparum* and *P. vivax* [18–22]. The competence of *An. superpictus* for *P. vivax* transmission has also been supported by an experimental infection study [23]. In contrast, its susceptibility to *P. falciparum* has never been directly tested. However, several authors have hypothesized that this species, belonging to the subgenus *Cellia*, which includes some of the most efficient African malaria vectors, could be permissive to Afrotropical strains of *P. falciparum* [9–12], unlike the refractoriness observed in vector species of the *An. maculipennis* complex [24, 25].

In Italy, *An. superpictus* exhibits a phenology typical of late summer mosquitoes, with adult populations usually peaking between August and September. Females are partially endophagic and exophilic, displaying opportunistic feeding behaviour preferentially shifted to large mammals when available. During the resting phase, they prefer warm indoor shelters and outbuildings [18]. As for larval habitats, the species generally breeds in residual pools formed in gravelly riverbeds when water flow decreases significantly in late summer and autumn. Larvae are typically found in small, slow-flowing pools on rocky riverbeds with scattered vegetation. The limited availability of such specific breeding sites, along with climatic variability and anthropogenic environmental changes, has significantly influenced the distribution and abundance of the species [9, 12, 18, 26, 27].

In the past, *An. superpictus* was collected in southern Italy including Sicily, predominantly along small river courses. It was less frequently found in central regions, only rarely recorded in the North [4, 16]. Currently, its range of distribution appears to be significantly restricted. In recent years, only sporadic records of few adult specimens have been documented in southern regions, such as Calabria, Basilicata and Apulia [11, 28, 29]. However, despite its apparent decline in abundance and range, *An. superpictus* should still be taken into consideration as a potential malaria vector, contributing to increase the receptivity of southern Italian localities [11, 29].

Considering the ongoing climatic and environmental changes, along with increased human mobility, entomological surveillance remains crucial for assessing the potential risk of malaria reintroduction in vulnerable areas. Data from field surveys contribute to strengthening the basic knowledge required to improve national prevention strategies. Within this framework, and as part of routine efforts to update residual anophelism data, entomological investigations were carried out between 2022 and 2024 in central Italy, specifically in the southern Maremma Plain (Grosseto province, Tuscany), a historically endemic region for malaria before its eradication. This study aims to describe the survey activities carried out and presents the findings obtained.

## Methods

### Study area

The study area is located in the southernmost part of the Maremma Plain countryside, specifically in the municipality of Magliano in Toscana (Grosseto province), approximately 20 kms from the Tyrrhenian Sea. The territory consists of modest hilly reliefs, none of which exceed 300–350 m above sea level (a.s.l.), along with some valleys traversed by the main river Albegna and its tributary streams. The Albegna riverbed is wide, gravelly and with riparian vegetation along the riverbank. It maintains a good level of cleanliness, flowing through a naturalistically significant environment included in the Maremma Park (SIR-SIC-ZPS code: 121 “Medio Corso del Fiume Albegna”—code Natura 2000 IT51A0021 and SIR-sir code B22 “Torrente Trasubbie” cod. IT5190103). Human impact on the area is limited, with contiguous arable fields and pastures. Typical settlement patterns consist of scattered houses and/or farms. In 2022, the residential population registered for the Municipality was 13.1 inhabitants per square kilometre. In this area, the climate is typically Mediterranean. During 2022, July and August were the hottest months. Maximum temperatures ranged from 32.5 °C in July to 21.6 °C in October; the average relative humidity was around 66%. In total 13 rainy days were recorded, with cumulative precipitation reaching 253.2 mm, of which 222.6 mm fallen in September (https://www.sir.toscana.it/consistenza-rete).

### Sampling sites

**Fig. 1:**
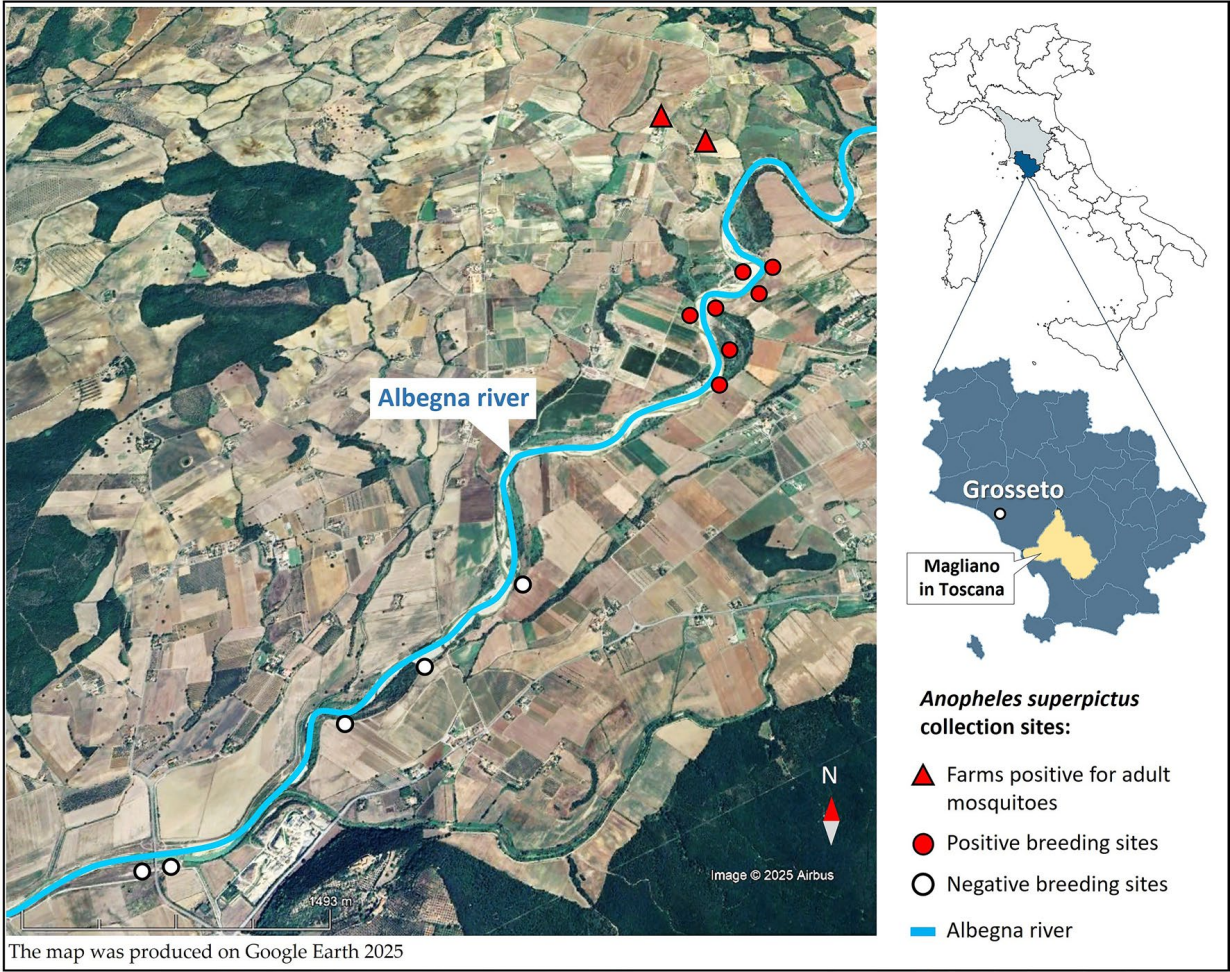
Map of the study area (Magliano in Toscana municipality, Grosseto province, Tuscany). Triangles represent the two farms where *Anopheles superpictus* adult samples were collected. Red dots indicate the positive larval breeding sites along the Albegna river, while white dots represent the negative ones

**Fig. 2:**
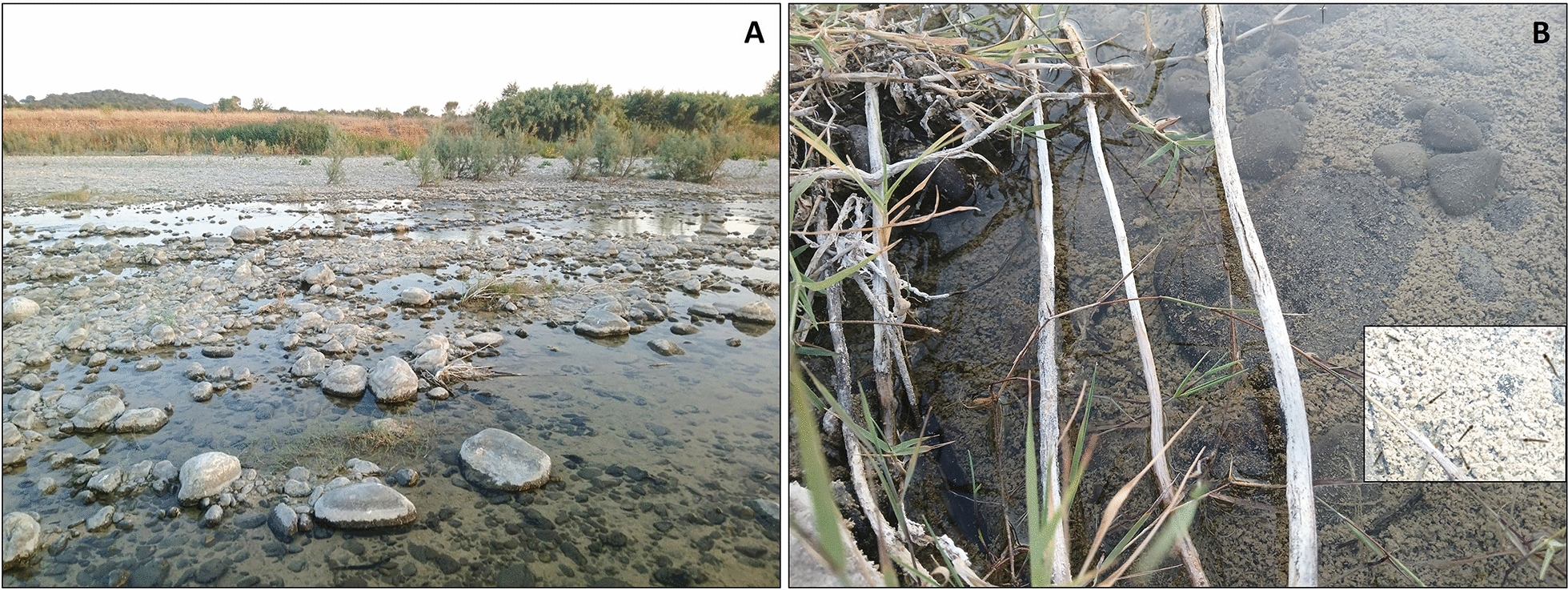
Larval breeding sites of *Anopheles superpictus* along the Albegna riverbed (Magliano in Toscana, Grosseto, Tuscany). **A** general view of the site. **B** close-up of a residual pool surrounded by low vegetation, showing in the right-hand box a detail of larval aggregation

Adult mosquito collections were carried out in two farms (42°35′21.9″ N; 11°22′09.8″ E), where domestic animals, particularly backyard poultry and stabled cattle are reared. Both sites are located below 300 m a.s.l. and lie near the course of the Albegna river (Fig. 1). For larva collections twelve potential breeding sites, situated within a radius of 1–5 kms from the farms, and along a slow-flowing section of the river characterized by shallow gravelly pools and aquatic vegetation, were investigated (Figs. 1 and 2).

### Mosquito collection

In 2022, adult mosquitoes were collected from July to October by using BG-PRO (Biogents^®^) and CDC light traps, placed at five different sites inside and outside the animal stables, operating from sunset to dawn. In addition, in September 2022, supplementary active sampling was conducted using electric aspirators to collect resting adults inside animal shelters and nearby tool premises. Larval sampling was also carried out along the Albegna riverside using standard 500 ml dippers, with 10–20 dips per site [30]. In September 2023 and 2024, only two snapshot surveys were conducted mid-monthly, using the same adult collection methods.

All adult specimens were stored at − 20 °C before their processing. Larvae were reared to the fourth instar for morphological identification, and then preserved in 70% ethanol.

### Specimen processing

Both larvae and adults were identified using dichotomous morphological keys of the Italian mosquito species [31–33]. Observations of morphological characters of the larvae and the adult specimens were performed in Phosphate-Buffered Saline solution (PBS) and at dry conditions respectively, under a stereomicroscope (Leica Stereozoom S9i). For species identification within the *An. maculipennis* complex specimens, gravid females were induced to lay eggs and the exochorion banding pattern was observed [34].

Furthermore, a subsample of *An. superpictus* were molecularly analysed for species confirmation by sequencing both the cytochrome c oxidase subunit 1 (*cox1*) gene and the ribosomal DNA internal transcribed spacer region 2 (*ITS2*). An 889-base pair fragment of the *cox1* gene was amplified by combining the forward primer LCO1490 [35] and the reverse primer (5′AAAAATTTTAATTCCAGTTGGAACAGC 3′) described in Kumar et al. [36]. The *ITS2* region was amplified using the 5.8S primer forward (5′-TGT GAA CTG CAG GAC ACA TG-3′) [37] and Hyr reverse primer (5′-GGG GTW GTC ACA CAT AAC TTG AGG-3) [38].

*Anopheles maculipennis* s.l. specimens were identified only by *ITS2* analyses, according to Marinucci et al. [39] protocol.

All PCR products were sequenced at Eurofins Genomics (Ebersberg, Germany) using the aforementioned primers. Resulting sequences were assembled using DS Gene v1.5 software (Accelrys Inc. 2003) and analysed using NCBI's Basic Local Alignment Search (BLAST) [40].

### Show data analysis

For the 2022 survey data, differences in mosquito species were assessed using the Shannon–Wiener diversity index (H), calculated as: H =  − ∑[(pi) × ln(pi)], where:

*∑:* sum

pi: proportion of individuals of i-th species in the whole community

ln: natural logarithm

pi = n/N (n*:* individual of a given species; N: total number of individuals in the community)

An H value of 0 indicates that only one species is present in the habitat; increasing H values correspond to greater species diversity.

The presence and abundance of anopheline species were compared using the non-parametric Mann–Whitney test (*U*-test). Differences were considered statistically significant at *P* < 0.05.

All statistical analyses were performed using R^®^ software (version 3.5.0).

## Results

Longitudinal surveys conducted from July to October 2022 resulted in the collection by traps of 1,106 mosquitoes, peaking abundance in August (*n* = 646) and September (*n* = 394). Specimens belonged to four genera: *Aedes*, *Anopheles*, *Culex* and *Culiseta* and nine species were identified through both morphological and molecular analyses (Table 1). The Shannon–Wiener diversity index (H) was calculated as 1.38, indicating a limited level of mosquito species diversity in the study area.

**Table 1:** Mosquito species collected using BG-PRO and CDC-light traps, from July to October 2022, in Magliano in Toscana municipality (Grosseto , Tuscany)

Month of collections	Mosquito species									Total per month
	*Aedes albopictus*	*Aedes caspius*	*Aedes detritus*	*Anopheles labranchiae*	*Anopheles superpictus*	*Culiseta annulata*	*Culiseta longiareolata*	*Culex pipiens s.l*	*Culex theileri*	
July	0	1	0	3	38	0	0	6	0	48
August	10	18	1	70	372	1	12	160	2	646
September	38	32	0	29	98	2	18	169	8	394
October	0	1	1	1	4	0	5	6	0	18
Total (percentage)	48 (4.3)	52 (4.7)	2 (0.2)	103 (9.3)	512 (46.3)	3 (0.3)	35 (3.2)	341 (30.8)	10 (0.9)	1106

Overall, among the Culicinae subfamily, *Culex pipiens* s.l. is the most abundant, accounting for 30.8% (260 females, 81 males,), followed by *Aedes caspius* (47 females, five males), *Ae. albopictus* (26 females, 22 males), *Culiseta longiareolata* (13 females, 22 males), *Cx. theileri* (seven females, three males), *Cs. annulata* (three females), *Ae. detritus* (two females).

**Fig. 3:**
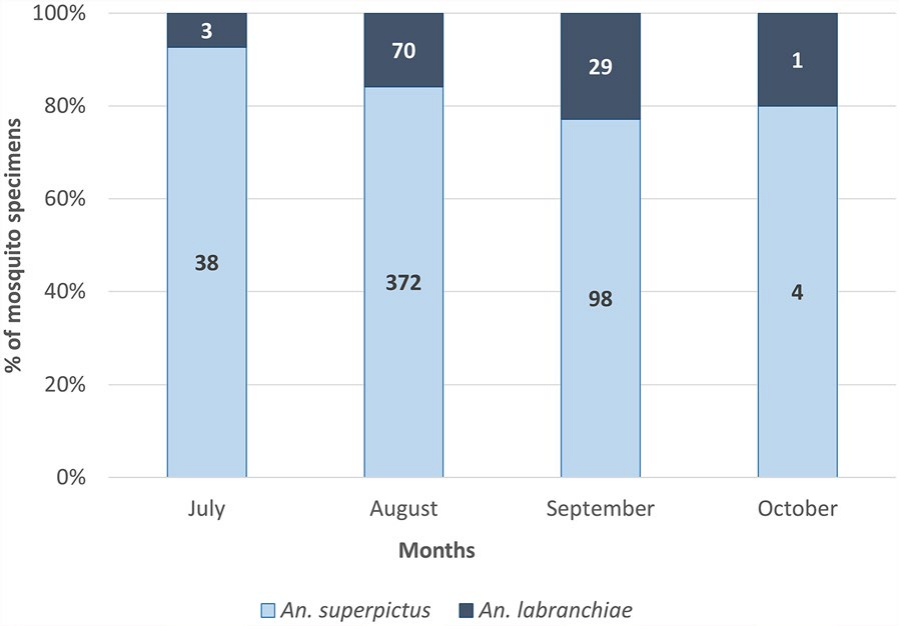
Monthly percentage distribution of *Anopheles superpictus* and *An. labranchiae* collected using BG-PRO and CDC light traps from July to October 2022 in two farms located in the municipality of Magliano in Toscana (Grosseto, Tuscany). Histogram columns represent the percentage distribution, with total specimen counts shown above each bar

Species belonging to the Anophelinae subfamily accounted for 56% of all specimens collected. *Anopheles superpictus* was the most abundant species, representing 46.3% of the total sample with 487 females, and 25 males, reaching its highest number in August (*n* = 372) and sharply declining in October (*n* = 4) (Table 1). *Anopheles labranchiae* was also recorded, with 9.3% of the total sample, including 97 females, and six males. The monthly percentage of the two anopheline species is shown in Fig. 3. The Mann–Whitney test (*U* test) compares the abundances of *An. superpictus* and *An. labranchiae* yielded *U* = 101 (*P* = 0.0003), indicating a statistically significant predominance of the former species.

The sex ratio of all collected mosquitoes was mostly biased toward females, particularly among *Anopheles* species, likely due to the reduced dispersal capacity of males from breeding sites.

Focusing on manual anopheline captures in September, a total of 30 engorged and gravid females were collected in a tool premise nearby a livestock shed, including 20 *An. superpictus* and 10 *An. labranchiae* specimens; consistent with trap collection results, *An. superpictus* was predominant, accounting for 67% of the specimens, compared to 33% for *An. labranchiae*.

**Fig. 4:**
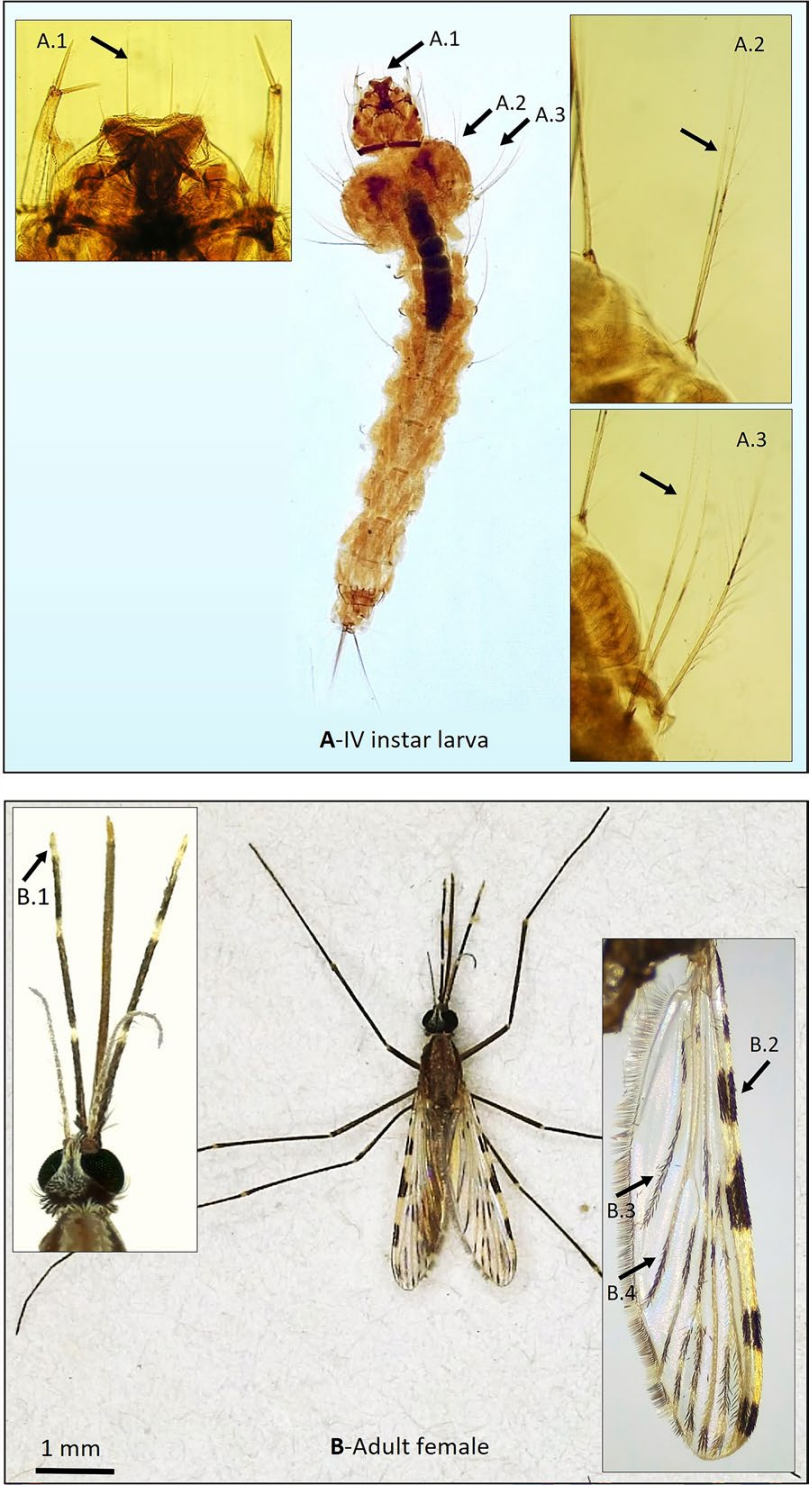
Morphological characteristics of *Anopheles superpictus* collected in the municipality of Magliano in Toscana (Grosseto, Tuscany). **A** IV instar larva. A.1: inner clypeal setae; A.2: long mesothoracic pleural setae, one simple, the other one with minute lateral branches, diagnostic for the species; A.3: long metathoracic pleural setae, both with lateral branches. **B** Adult female. B.1: Head with white scales at the apex of the maxillary palpi; B.2: colour pattern of the costal vein (subgenus *Cellia*); B.3: colour pattern of the anal vein; B.4: dark and pale spots on the cubital vein

Among the twelve breeding sites investigated in the Albegna riverbed area, selected based on accessibility and proximity to farms, seven (58%) tested positive for *An. superpictus* larvae. Overall, 50 specimens were collected, with mean densities ranging from 0.1 to 0.2 larvae per dip (Fig. 2). All adults and larvae of the *An. superpictus* were morphologically identified based on species-specific characters, as illustrated in Fig. 4.

As support of morphological identification, molecular analyses were carried out on 4% of the adult *An. superpictus* specimens (*n* = 20 out of 512). All *cox1* and *ITS2* sequences obtained from the 20 specimens were identical, with no observed nucleotide variation. One representative sequence for each marker was deposited in GenBank. The accession numbers of the newly generated sequences acquired in this study are PQ570580 (*ITS2*) and PQ569434 (*cox1*). Moreover, BLAST analysis of *cox1* and *ITS2* obtained sequences showed a > 99% nucleotide homology with *An. superpictus* available sequences in GenBank. In particular, the *cox1* consensus fragment of 889 nucleotides exhibited 99.10% identity with a sequence from a specimen collected in Greece (MT993498). When considering only the *cox1* region (658 nucleotides), that would be obtained by amplification with the most utilized LCO1480-HCO2198 primers [35], identities ranged from 99.09 to 98.94% when compared to several sequences derived from samples collected in Tajikistan (JX255703-JX255708). Additionally, *ITS2* sequences exhibited 100% identity with those obtained from *An. superpictus* from several WHO European and Eastern Mediterranean countries, including Greece (FJ526598), Uzbekistan (AY515163), Afghanistan (EU482199), Iran (KF483835) and Pakistan (DQ487149).

**Fig. 5:**
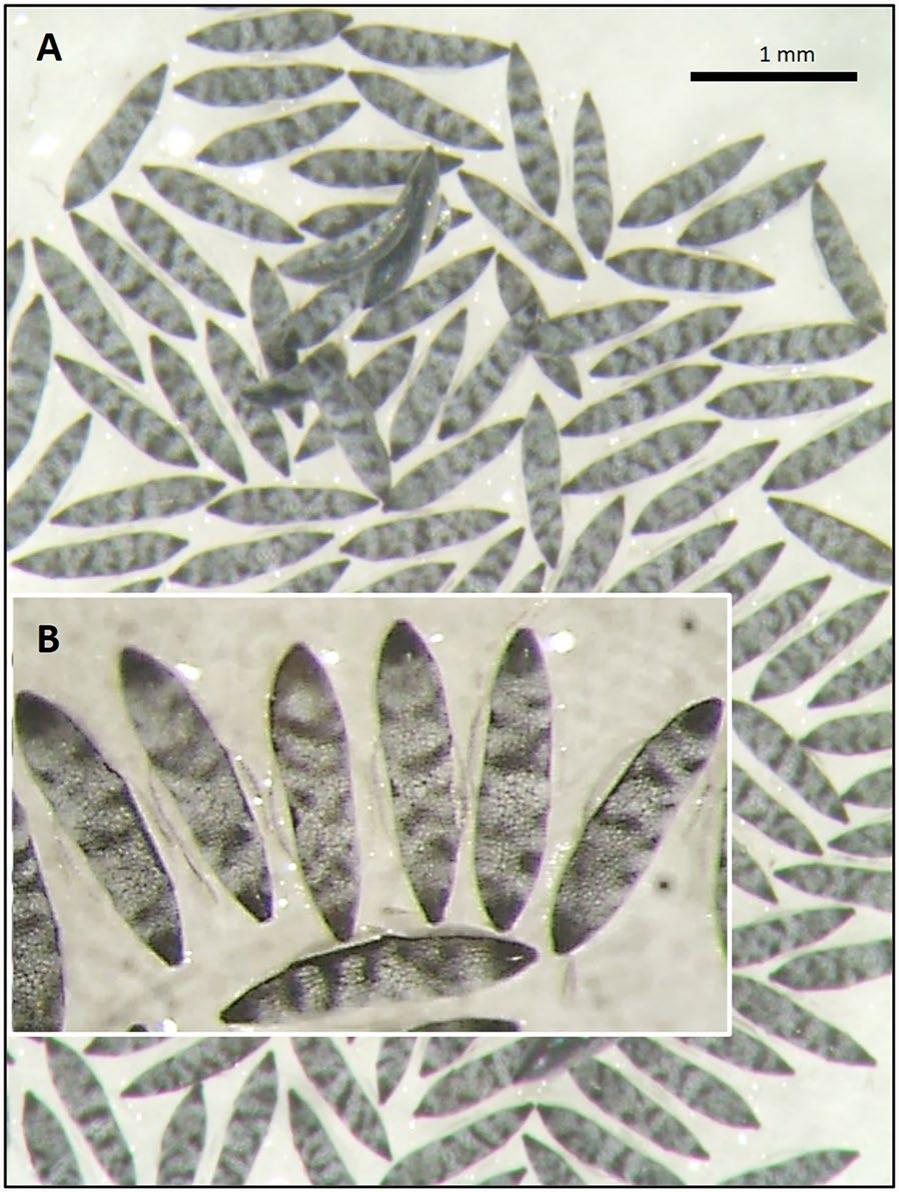
Oviposition of *Anopheles labranchiae* collected in Magliano in Toscana municipality (Grosseto, Tuscany). **A**: General view of the egg-banding pattern, showing the egg exochorion with wedge-shaped dark marks on a pale background. **B**: Magnified view of the eggs, showing short and narrow floats

Specimens included in the *An. maculipennis* s.l. were identified as *An. labranchiae* through egg pattern observations (Fig. 5). The species was also confirmed by *ITS2* sequencing as the only member of the complex detected in the area. The GenBank accession number of *An. labranchiae ITS2* sequence is PV798893.

In September 2023 and 2024, two snapshot surveys were conducted using CDC light traps to confirm the continued presence of *An. superpictus* in the area. During these surveys, 15 and 32 females of this species were collected, along with three and five *An. labranchiae* specimens.

## Discussion

This study, carried out in the period 2022–2024, documented new field records of *An. superpictus* in sites located in Magliano in Toscana municipality (Grosseto province), at the southern border of Maremma Plain. Notably, no historical or recent data on anopheline mosquitoes were previously available for this area. Moreover, the last records of the *An. superpictus* in inland localities in Grosseto province date back nearly a century, during anti-malaria control plans implemented at that time [41–43].

In the 2022 longitudinal surveys (July–October), *An. superpictus* was recorded at consistently high densities. It emerged as the most abundant mosquito species captured by both traps and manual collections. The application of the Shannon–Wiener diversity index provides a quantitative measure supporting the observed species pattern in the study area (Table 1). However, the low H value, which confirms the dominance of *An. superpictus*, should be interpreted with caution due to the limited spatial and temporal coverage of the sampling.

Larval sampling further enabled the characterization of its natural breeding sites, an achievement unreported in recent decades. The ecological characteristics of the study area appear particularly suitable for the development of *An. superpictus*. The gravelly bed of the Albegna river, the absence of dense vertical vegetation, and the presence of shallow water bodies, offering larvae protection from larvivorous fish, correspond closely with the known habitat preferences of the species (Fig. 2).

The snapshot surveys conducted in September 2023 and 2024 confirmed the continued presence of the species, although the number of specimens collected was markedly lower than in the same period of 2022. This reduction may partly reflect the limitations of single-time-point sampling methods. However, meteorological factors likely played a role as well.

*An. superpictus* populations tend to fluctuate in response to river water levels: years with frequent summer showers or prolonged droughts may hinder the formation of suitable breeding sites, leading to reduced mosquito abundance. Likewise, the species’ seasonal trend is variable. As river levels decline during mid-summer and isolated pools occur, optimal breeding conditions emerge, allowing population growth that typically peaks between September and October [18]. From an epidemiological standpoint, such year-to-year fluctuations in vector density may influence the receptivity of an area. In settings where malaria parasites circulate at low levels, increased densities of *An. superpictus* may enable the species to spread into nearby areas previously considered malaria-free, potentially resulting in malaria outbreaks [12, 18, 44].

In 2022, *An. superpictus* reached its peak abundance in August and September, the two wettest months of the summer. Total rainfall during this period amounted to 232.2 mm, in stark contrast to 57.8 mm in 2023 and 70.8 mm in 2024. Nevertheless, the snapshot surveys confirmed that even in years with scarce precipitation, when residual pools are reduced, the species remains present in the study area, albeit at lower densities.

In both surveyed farms, adult specimens of *An. superpictus* were found in sympatry with *An. labranchiae*. Together, these two anopheline species accounted for more than half of the mosquito fauna collected in the area. However, *An. labranchiae* was recorded at significantly lower densities compared to *An. superpictus*.

Larval habitats for *An. labranchiae* could not be identified in the study area, as no larvae were found during field sampling. This former malaria vector, frequently recorded at notable densities in several areas of Grosseto province [25, 42, 45, 46], typically exploits a wide variety of larval habitats. They can range from coastal salt marshes to artificial water bodies created by human activities, such as rice paddies and irrigation canals, demonstrating the remarkable ecological plasticity of the species [46, 47]. Although the Albegna riverbed offers ideal breeding conditions for *An. superpictus*, certain sections of the river may also provide suitable environments for *An. labranchiae*, even if this could not be confirmed through larval sampling.

To support species identification, molecular analyses were also conducted on *An. superpictus* specimens collected in Magliano in Toscana. Sequence analysis of the *cox1* and *ITS2* genes revealed a high degree of similarity with multiple haplotypes available in GenBank, originating from populations across several European and Eastern Mediterranean countries. In some of these countries, the species is recognized as a competent malaria vector [19–22, 44]. Furthermore, from a phylogenetic perspective, several studies have identified *An. superpictus* as a species complex, including at least three closely related taxa, each potentially playing a different role in malaria transmission [14, 48, 49]. Nevertheless, the taxonomic status within this complex has so far remained unresolved [13, 15].

## Conclusions

This study, although limited by surveys conducted on a small local scale and on a seasonal basis, contributes substantially to the current knowledge of the presence and distribution of *An. superpictus* in Italy, providing new and consistent field records of a species rarely reported in recent decades. It also adds valuable evidence to entomological data at the European level [17].

Moreover, the findings may serve as a starting point for future research. Broader investigations across central Italy, combined with population genetic analysis, will be essential to clarify the current epidemiological relevance of the species.

Investigations in Magliano in Toscana revealed that *An. superpictus* could reach high densities in the study area and, together with the stable presence of *An. labranchiae*, these findings highlight the importance of regular, targeted monitoring to assess changes in species abundance and distribution over time. These two former malaria vectors may gradually disperse throughout the territory, depending on the availability of suitable larval habitats, potentially leading to dynamic changes in distribution and an evolving receptivity in vulnerable areas.

## Data Availability

Data supporting the main conclusions of this study are included in the manuscript.

## Abbreviation

PBS: Phosphate-Buffered Saline solution
PCR: Polymerase Chain Reaction

## References

[1] World Health Organization. World malaria report 2024. Geneva: WHO; 2024. https://www.who.int/teams/global-malaria-programme/reports/world-malaria-report-2024. Accessed 26 Jun 2025.

[2] European centre for disease prevention and control. Malaria—annual epidemiological report for 2022. Stockholm: ECDC; 2024. https://www.ecdc.europa.eu/en/publications-data/malaria-annual-epidemiological-report-2022. Accessed 26 Jun 2025.

[3] European Centre for Disease Prevention and Control. *Anopheles maculipennis* s.l.—current known distribution: October 2023. Stockholm: ECDC; 2023. https://www.ecdc.europa.eu/en/publications-data/anopheles-maculipennis-sl-current-known-distribution-october-2023. Accessed 26 Jun 2025.

[4] Majori G. Short history of malaria and its eradication in Italy with short notes on the fight against the infection in the Mediterranean basin. Mediterr J Hematol Infect Dis. 2012;4:e2012016. 22550561 10.4084/MJHID.2012.016 PMC3340992

[5] Romi R, Pierdominici G, Severini C, Tamburro A, Cocchi M, Menichetti D, et al. Status of malaria vectors in Italy. J Med Entomol. 1997;34:263–71. 10.1093/jmedent/34.3.263. 9151488 10.1093/jmedent/34.3.263

[6] Bietolini S, Candura F, Coluzzi M. Spatial and long-term temporal distribution of the *Anopheles maculipennis* complex species in Italy. Parassitologia. 2006;48:581–608. 17688180

[7] Raele DA, Severini F, Toma L, Menegon M, Boccolini D, Tortorella G, et al. *Anopheles sacharovi* in Italy: first record of the historical malaria vector after over 50 years. Parasit Vectors. 2024;17:182. 10.1186/s13071-024-06252-2. 38600589 10.1186/s13071-024-06252-2 PMC11005165

[8] Raffaele G. Note sull’eradicazione della malaria in Italia. Riv Malariol. 1964;43:1–27. 14192246

[9] Sabatini A, Coluzzi M, Boccolini D. Field studies on inversion polymorphism in *Anopheles superpictus* from southern Italy. Parassitologia. 1989;31:69–87. 2487896

[10] Romi R. *Anopheles labranchiae*, an important vector in Italy, and other potential malaria vectors in southern Europe. Eur Mosq Bull. 1999;4:8–10.

[11] Romi R, Sabatinelli G, Majori G. Could malaria reappear in Italy? Emerg Infect Dis. 2001;7:915–9. 10.3201/eid0706.010601. 11747716 10.3201/eid0706.010601 PMC2631915

[12] Jetten TH, Takken W. Anophelism without malaria in Europe: a review of the ecology and distribution of the genus *Anopheles *in Europe. 3rd ed. Wageningen: Wageningen Agricultural University; 1994. p. 69.

[13] Sinka ME, Bangs MJ, Manguin S, Coetzee M, Mbogo CM, Hemingway J, et al. The dominant *Anopheles* vectors of human malaria in Africa, Europe and the Middle East: occurrence data, distribution maps and bionomic precis. Parasit Vectors. 2010;3:117. 10.1186/1756-3305-3-117. 21129198 10.1186/1756-3305-3-117 PMC3016360

[14] Vatandoost H, Hanafi-Bojd AA, Raeisi A, Abai MR, Nikpour F. Bioecology of dominant malaria vector, *Anopheles superpictus* s.l. (Diptera: Culicidae) in Iran. J Arthropod Borne Dis. 2018;12:196–218. 30584544 PMC6297731

[15] World Health Organization. Mosquitoes of the genus *Anopheles* in countries of the WHO European Region having faced a recent resurgence of malaria: regional research project, 2003–2007. Copenhagen: WHO Regional Office for Europe; 2008. Report No.: E922010. https://iris.who.int/handle/10665/107914.

[16] Bertola M, Mazzucato M, Pombi M, Montarsi F. Updated occurrence and bionomics of potential malaria vectors in Europe: a systematic review (2000–2021). Parasit Vectors. 2022;15:88. 10.1186/s13071-022-05204-y. 35292106 10.1186/s13071-022-05204-y PMC8922938

[17] European Centre for Disease Prevention and Control. *Anopheles superpictus* – current known distribution: October 2023. Stockholm: ECDC; 2023. https://www.ecdc.europa.eu/en/publications-data/anopheles-superpictus-current-known-distribution-october-2023.

[18] World Health Organization. Malaria vectors and approaches to their control in malaria affected countries of the WHO European Region: proceedings of a regional meeting on vector biology and control; 2001 May 3–5; Almaty, Kazakhstan. Copenhagen: WHO Regional Office for Europe; 2001. Report No.: EUR/01/5027499. https://iris.who.int/handle/10665/107428.

[19] Faulde MK, Hoffmann R, Fazilat KM, Hoerauf A. Malaria reemergence in northern Afghanistan. Emerg Infect Dis. 2007;13:1402–4. 10.3201/eid1309.061325. 18252122 10.3201/eid1309.061325 PMC2857272

[20] Habirov Z, Manilova E, Kadamov D, Komilova S, Harbach RE. ELISA Incrimination of *Anopheles superpictus* and *Anopheles hyrcanus* (Diptera: Culicidae) as vectors of *Plasmodium vivax* (Haemosporida: Plasmodiidae) in Tajikistan. J Med Entomol. 2013;50:1298–302. 10.1603/ME13110. 24843935 10.1603/me13110

[21] Kondrashin AV, Sharipov AS, Kadamov DS, Gasimov E, Baranova AM, et al. Elimination of *Plasmodium falciparum* malaria in Tajikistan. Malar J. 2017;16:226. 10.1186/s12936-017-1861-5. 28558764 10.1186/s12936-017-1861-5 PMC5450305

[22] Yavaşoglu Sİ, Yaylagül EÖ, Akıner MM, Ülger C, Çağlar SS, Şimşek FM. Current insecticide resistance status in *Anopheles sacharovi* and *Anopheles superpictus* populations in former malaria endemic areas of Turkey. Acta Trop. 2019;193:148–57. 10.1016/j.actatropica.2019.02.003. 30742803 10.1016/j.actatropica.2019.02.003

[23] Kasap H. Comparison of experimental infectivity and development of *Plasmodium vivax* in *Anopheles sacharovi* and *An.**superpictus* in Turkey. Am J Trop Med Hyg. 1990;42:111–7. 10.4269/ajtmh.1990.42.111. 2180330 10.4269/ajtmh.1990.42.111

[24] Ramsdale CD, Coluzzi M. Studies on the infectivity of tropical African strains of *Plasmodium falciparum* to some southern European vectors of malaria. Parassitologia. 1975;17:39–48. 787897

[25] Romi R, Boccolini D, Vallorani R, Severini F, Toma L, Cocchi M, et al. Assessment of the risk of malaria re-introduction in the Maremma plain (Central Italy) using a multi-factorial approach. Malar J. 2012;11:98. 10.1186/1475-2875-11-98. 22463387 10.1186/1475-2875-11-98 PMC3395869

[26] Sabatini A, Terranova F, Cianchi R, Coluzzi M. Ricerche sull’anofelismo delle fiumare della costa ionica calabrese. Parassitologia. 1981;23:245–9.

[27] Hertig E. Distribution of *Anopheles* vectors and potential malaria transmission stability in Europe and the Mediterranean area under future climate change. Parasit Vectors. 2019;12:18. 10.1186/s13071-018-3278-6. 30621785 10.1186/s13071-018-3278-6 PMC6325871

[28] Fares F, Mancini G, Santilli A, Goffredo M, Latorre L, Cafiero MA, et al. Ritrovamento di *Anopheles* (Cellia) *superpictus* in Basilicata. Biol Ital. 2012;42:34–8.

[29] Raele DA, Severini F, Boccolini D, Menegon M, Toma L, Vasco I, et al. Entomological surveillance in former malaria-endemic areas of southern Italy. Pathogens. 2021;10:1521. 10.3390/pathogens10111521. 34832676 10.3390/pathogens10111521 PMC8619560

[30] European Centre for Disease Prevention and Control. Guidelines for the surveillance of native mosquitoes in Europe. Stockholm: ECDC; 2014. 111 p. https://www.ecdc.europa.eu/en/publications-data/guidelines-surveillance-native-mosquitoes-europe.

[31] Sabatini A, Coluzzi M. Corso di aggiornamento in malariologia. Rome: Istituto Superiore di Sanità; 1981. 64 p.

[32] Romi R, Pontuale G, Sabatinelli G. Le zanzare italiane: generalità e identificazione degli stadi preimaginali (Diptera, Culicidae). Fragm Entomol. 1997;29:1–141.

[33] Severini F, Toma L, Di Luca M, Romi R. Le zanzare italiane: Generalità e identificazione degli adulti (Diptera, Culicidae). Fragm Entomol. 2009;41:213–72.

[34] Angelucci A. Tavole sinottiche sugli anofelini italiani. ACIS Monogr Ann Sanita Pubblica. 1955;1:1–19.

[35] Folmer O, Black M, Hoeh W, Lutz R, Vrijenhoek R. DNA primers for amplification of mitochondrial cytochrome c oxidase subunit I from diverse metazoan invertebrates. Mol Mar Biol Biotechnol. 1994;3:294–9. 7881515

[36] Kumar NP, Rajavel AR, Natarajan R, Jambulingam P. DNA barcodes can distinguish species of Indian mosquitoes (Diptera: Culicidae). J Med Entomol. 2007;44:1–7. 17294914 10.1603/0022-2585(2007)44[1:dbcdso]2.0.co;2

[37] Porter CH, Collins FH. Species-diagnostic difference in a ribosomal DNA internal transcribed spacer from the sibling species *Anopheles freeborni* and *Anopheles hermsi* (Diptera: Culicidae). Am J Trop Med Hyg. 1991;45:271–9. 1877723 10.4269/ajtmh.1991.45.271

[38] Severini C, Menegon M, Di Luca M, Abdullaev I, Majori G, Razakov SA, et al. Risk of *Plasmodium vivax* malaria reintroduction in Uzbekistan: genetic characterization of parasites and status of potential malaria vectors in the Surkhandarya region. Trans R Soc Trop Med Hyg. 2004;98:585–92. 10.1016/j.trstmh.2004.01.003. 15289095 10.1016/j.trstmh.2004.01.003

[39] Marinucci M, Romi R, Mancini P, Di Luca M, Severini C. Phylogenetic relationships of seven palearctic members of the *maculipennis* complex inferred from *ITS2* sequence analysis. Insect Mol Biol. 1999;8:469–80. 10.1046/j.1365-2583.1999.00140.x. 10634971 10.1046/j.1365-2583.1999.00140.x

[40] Camacho C, Coulouris G, Avagyan V, Ma N, Papadopoulos J, Bealer K, et al. BLAST+: architecture and applications. BMC Bioinform. 2009;10:421. 10.1186/1471-2105-10-421. 10.1186/1471-2105-10-421 PMC2803857 20003500

[41] Galamini A. La fauna anofelica della Maremma grossetana e la lotta invernale contro la malaria. Ann d’Igiene. 1923;33:627–39.

[42] Bettini S. L’anofelismo e la malaria in Maremma. Quaderni Associazione Ricerche Ambiente (Orbetello, Grosseto). Assoc Ric Ambiente. 1997;97:1.

[43] Bueti S, Corti M. La malaria nella Maremma grossetana: una malattia dagli aspetti sociali. Med Secoli. 1998;10:541–55. 11623701

[44] Hanafi-Bojd AA, Sedaghat MM, Vatandoost H, Azari-Hamidian S, Pakdad K. Predicting environmentally suitable areas for *Anopheles superpictus* Grassi (s.l.), *Anopheles maculipennis* Meigen (s.l.) and *Anopheles sacharovi* Favre (Diptera:Culicidae) in Iran. Parasit Vectors. 2018;11:382. 10.1186/s13071-018-2973-7. 29970145 10.1186/s13071-018-2973-7 PMC6029181

[45] Di Luca M, Boccolini D, Severini F, Toma L, Barbieri FM, Massa A, et al. A 2-year entomological study of potential malaria vectors in central Italy. Vector Borne Zoonotic Dis. 2009;9:703–11. 10.1089/vbz.2008.0129. 19485768 10.1089/vbz.2008.0129

[46] Boccolini D, Toma L, Di Luca M, Severini F, Cocchi M, Bella A, et al. Impact of environmental changes and human-related factors on the potential malaria vector, *Anopheles labranchiae* (Diptera: Culicidae), in Maremma, Central Italy. J Med Entomol. 2012;49:833–42. 10.1603/me11252. 22897043 10.1603/me11252

[47] Bettini S, Gradoni L, Cocchi M, Tamburro A. Rice culture and *Anopheles labranchiae* in Central Italy. WHO/Malaria. 1978;78:1–6.

[48] Oshaghi MA, Shemshad K, Yaghobi-Ershadi MR, Pedram M, Vatandoost H, Abaie MR, et al. Genetic structure of the malaria vector *Anopheles superpictus* in Iran using mitochondrial cytochrome oxidase (COI and COII) and morphologic markers: a new species complex? Acta Trop. 2007;101:241–8. 10.1016/j.actatropica.2007.02.006. 17367742 10.1016/j.actatropica.2007.02.006

[49] Oshaghi MA, Yaghobi-Ershadi MR, Shemshad K, Pedram M, Amani H. The *Anopheles superpictus* complex: introduction of a new malaria vector complex in Iran. Bull Soc Pathol Exot. 2008;101:429–34. 10.3185/pathexo3245. 19192616

